# Personal identification via matching of curved multiplanar computed tomography reconstructions and panoramic radiographs

**DOI:** 10.1371/journal.pone.0337989

**Published:** 2025-12-04

**Authors:** Linus Woitke, Ulf Teichgräber, Gita Mall, Andreas Heinrich

**Affiliations:** 1 Department of Radiology, Jena University Hospital – Friedrich Schiller University, Jena, Thuringia, Germany; 2 Institute of Forensic Medicine, Jena University Hospital – Friedrich Schiller University, Jena, Thuringia, Germany; Universiti Sains Malaysia, MALAYSIA

## Abstract

Computer vision (CV)-based personal identification enables automated matching of recent radiological images with clinical databases to identify unknown individuals. This study aimed to assess whether a panoramic radiograph (PR)-like image reconstructed from computed tomography (CT) data using curved multiplanar reconstruction could enable CV-based personal identification using a PR database. A method was developed to automatically generate PR-like images with adjustable parameters, based on 50 CT examinations including the jaw region (38.64 ± 16.72 years; 17 females, 33 males), allowing for variations such as tooth rotations. Systematic modification of parameters enabled the generation of different representations to determine optimal settings for a large number of individuals. Multiple PR-like images per identity were tested against a PR database containing 82,036 PRs from 43,379 individuals. Utilizing the most effective individual parameter settings, 72% (36/50) of individuals were correctly identified at rank 1, 82% (41/50) at rank 10, and 96% (48/50) at rank 100 – out of 43,379 possible individuals. The rank describes the position of the matched image in a list sorted after a descending similarity score. When the optimal parameters were applied to a larger number of individuals, the identification rates were 50% (25/50) at rank 1, 64% (32/50) at rank 10, and 78% (39/50) at rank 100. In conclusion, CV demonstrates potential for personal identification by comparing automatically generated PR-like images with a large PR database.

## Introduction

In emergency departments, unidentified individuals may be admitted following accidents, natural disasters, or other critical events [1,2]. In such situations, computed tomography (CT) is frequently used as a diagnostic tool [3]. The development of an automated method for identifying these individuals by utilizing CT data obtained in emergencies and matching it with clinical databases may be a promising approach. One such method was developed by Heinrich and is referred to as computer vision (CV)-based personal identification [4–8]. This approach utilizes CV, a technique that emulates human vision by detecting unique anatomical features in imaging data and matching them against a reference database to identify potential identity matches. Whilst it is not feasible for humans to manually compare thousands of images within a reasonable time frame, CV can process large datasets efficiently and offer valuable leads in the identification process. CV-based personal identification has been successfully applied to both panoramic radiograph (PR)-to-PR [4,5] and CT-to-CT [6–8] comparisons. However, it remains unclear whether this approach is effective across different modalities, particularly whether PR-like images reconstructed from CT data can be matched to a large clinical PR database. These PR-like images are generated using curved multiplanar reformatting (curved MPR), an advanced imaging technique that allows the reconstruction of planar views along curved anatomical structures, such as the dental arch, from volumetric CT datasets. This facilitates the creation of PR-like images that closely resemble PRs, enabling direct comparison. This is of particular relevance given the fact that PRs are far more common than CT scans in the general population. If feasible, this cross-modality matching approach could significantly enhance the method’s applicability by enabling the identification of probable identities even in the absence of prior CT data. The clinical relevance of this technique extends well beyond forensic applications. In clinical emergencies, rapid and reliable identification offers substantial benefits. Such benefits include facilitating timely access to a patient’s medical records, supporting informed treatment decisions, enabling earlier contact with relatives, and, in some cases, helping to clarify the circumstances of the incident. Furthermore, in a forensics context, the implementation of a reliable automated identification method could assist forensic experts in the selection of suitable reference materials for legally secure identification. To date, only a few studies have investigated the comparability of PR-like images from multidetector CT data with actual PRs, typically using manual or semi-automated analysis of small sample sizes [9–11]. In 2007, Tohnak et al. [9] demonstrated that CT data can be used to reconstruct PR-like images using semi-automated computer algorithms with user-defined inputs. In two cases, visual comparison with PRs showed reduced distortion, blurring, and superimposition. Fujimoto et al. [10] compared PR-like images of two living individuals with 100 reference PRs using Procrustes analysis and correlation coefficients. For this, anatomical landmarks on the alveolar ridges and tooth sockets were manually marked on both the PR-like images and the PRs. They identified the best matches through statistical shape analysis and visual inspection, ultimately confirming identities with a precise 6-segment method. Vincent and Smythe [11] assessed various PR-like images, with an experienced forensic odontologist manually grading their similarity to PRs using a standardized 5-point scale to determine the most suitable reconstruction for identification purposes. While these studies demonstrated the feasibility of CT-to-PR comparisons, the process relied heavily on manual inspection or the placement of anatomical landmarks, which is impractical for large-scale datasets. In contrast, CV-based personal identification is fully automated and scalable, enabling efficient analysis of tens of thousands of images without human intervention. To our knowledge, no previous study has applied such a fully automated, large-scale CV-based approach to cross-modality matching between PR-like images and PRs.

This study addresses this gap by developing an automated method for generating PR-like images from multidetector CT data. Validation was performed by assessing the robustness and accuracy of cross-modality matching between PR-like images and a large clinical PR database containing 43,379 identities. The hypothesis is that CV-based personal identification can be successfully applied across modalities using this approach. If successful, this method could significantly broaden the applicability of CV-based personal identification. It would enable identification even without prior CT reference data and provide a reliable cross-modality tool in emergency and forensic contexts.

## Materials and methods

### Study design

All methods used in the study were approved by the local ethics committee of the Jena University Hospital (registration number 2019–1505-MV) and were carried out in accordance with relevant guidelines and regulations. As this was a retrospective analysis of routine work, written informed consent was waived by the ethics committee. The imaging data were exported from the clinical image database on 1 November 2023 and pseudonymized prior to analysis to protect participant identities. For this purpose, each individual was assigned a unique pseudonymized identifier, allowing data linkage without revealing identifiable information. No identifiable information was available during data analysis.

In this retrospective study, 50 CT examinations including the jaw region (GE Revolution, voltage: 100–120 kVp, tube current: 108.84 ± 61.13 mA, slice thickness: 0.625 mm, spacing between slices: 28 × 0.3125 mm and 22 × 0.625 mm) were randomly selected from a pool of 577 individuals identified from the clinical image database. All selected individuals had both a CT scan covering the jaw region and a corresponding PR available. The CT data were reconstructed using deep learning and iterative reconstruction methods. The timing of the PR or CT examination did not affect the selection. For the 50 individuals (age range 13–82 years, mean age 38.64 ± 16.72 years; 17 females, 33 males), CT scans were acquired between November 2010 and March 2023. The cases were selected based on the following inclusion criteria: at least 12 intact teeth in total across both jaws (no specific teeth were required), the absence of significant metal artifacts within the oral cavity, no strong rotation along the anteroposterior axis, and the availability of at least one corresponding PR for each individual. The selected cases reflect a wide spectrum of demographic and dental variability present in the larger PR database, including age, gender, number of teeth (26.38 ± 5.02), small fillings (mean 3.14 ± 2.91, range 0–12), crowns/large fillings (mean 0.58 ± 0.95, range 0–4), implants/braces (mean 0.24 ± 0.74, range 0–4), and varying degrees of tooth overlap in the CT images (no overlap: 20, partial: 17, yes: 13). Age distribution of the cohort included 9 individuals aged 13–24 years, 16 aged 25–34 years, 16 aged 35–54 years, and 9 aged 55–82 years. Despite the limited size, this set is sufficient for proof of concept evaluation of the robustness and generalizability of the proposed automated CV-based personal identification method. Detailed information on the test cases, including CT slice spacing and dental status, is provided in the S1 Table.

### Curved multiplanar reconstruction

**Fig 1:**
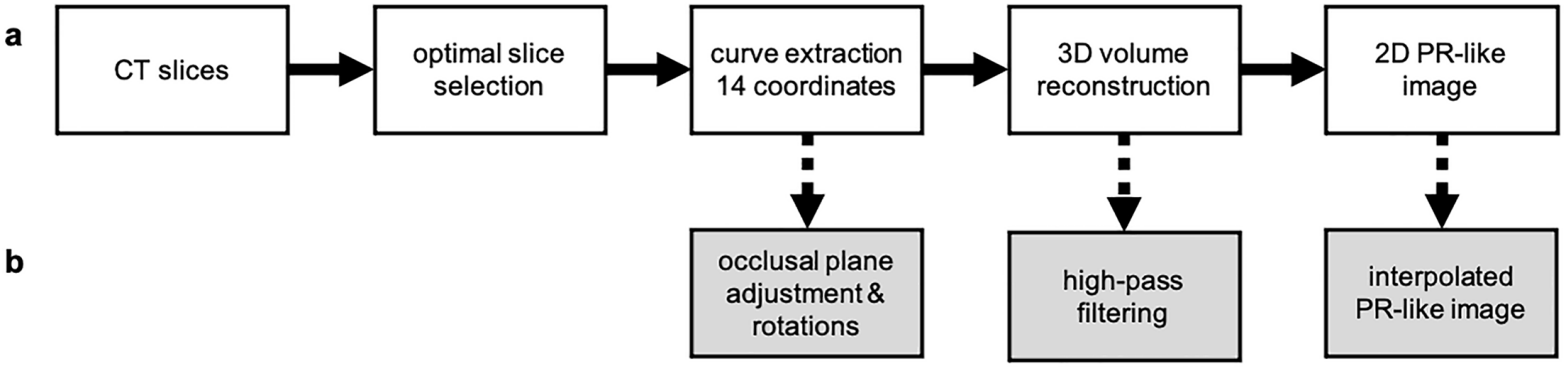
Flowchart of the automated reconstruction process. The diagram illustrates the steps involved in generating a PR-like image from a CT series, from left to right: a) standard reconstruction, b) additional optional steps to increase variability, including application of different rotations, high-pass filtering, or interpolation.

For each individual, a single CT series including the jaw region (i.e., a stack of 2D slices forming a 3D volume) serves as input. From this single 3D CT volume, multiple PR-like images can be automatically generated by defining different curved MPR volumes within the CT data, effectively simulating various views of jaw rotations. The reconstruction process involves several steps: localization of tooth coordinates by capturing the lower jaw, adjustment of these coordinates for different rotational perspectives, generation of a 3D volume from the coordinates, soft tissue filtering, calculation of a 2D PR-like image, and final interpolation to achieve the desired resolution (Fig 1). Each step is explained in more detail below and visualized in the S1–S3 Figs. Although there are no strict requirements for the CT examination parameters, thin, contiguous or overlapping slices are advantageous for improved reconstruction quality.

#### Tooth coordinate localization (standard PR-like image).

**Fig 2:**
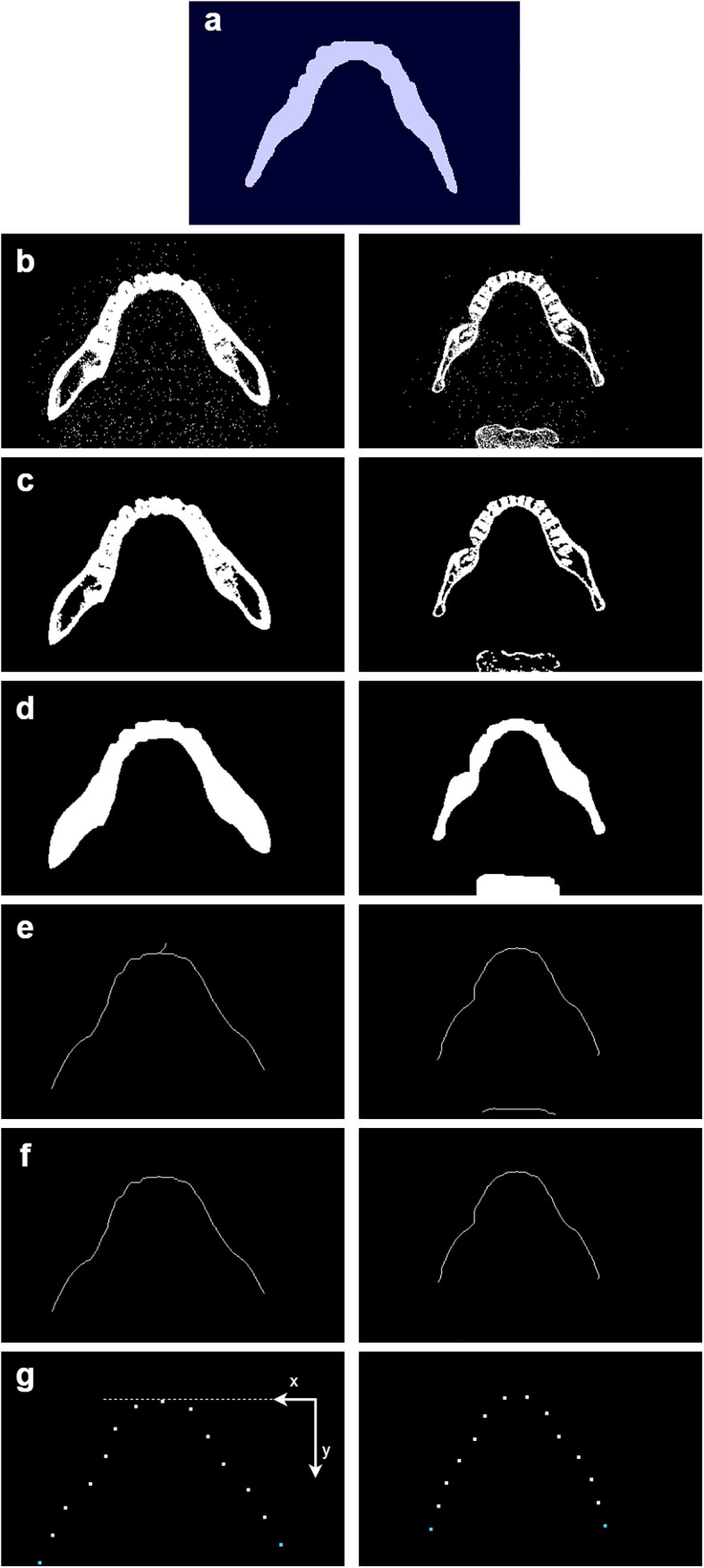
Steps in the extraction of 14 coordinates from the CT slices for the curved MPR. It shows the step-by-step transformation for two examples on the left and right sides: a) a binary mask of the lower dental arch is shown, which is compared with the binarized CT images to obtain the best slice containing the lower jaw, as shown in b). The image is then cleaned in c) using morphological filtering with an *opening* operation to remove small-scale noise. Subsequent to this, morphological filtering with a *closing* operation is applied, where gaps or inconsistencies in the outline are filled to improve the overall shape of the jaw, as shown in d). In e), the image is skeletonized by creating a line along the center of the lower jaw. Protruding paths are then eliminated using graph-based optimization algorithms, as demonstrated in f). Along this optimized curve, 12 evenly spaced coordinates are extracted, and the curve is extended with an additional coordinate at each end, represented by the blue points in g).

Initially, the CT slices are automatically analyzed using Python (version 3.9.10) and OpenCV (version 4.9.0) to identify the slice with the best representation of the lower dental arch. To achieve this, the Digital Imaging and Communications in Medicine (DICOM) images are binarized using a high-pass filter with an intensity threshold of 500 HU, thereby highlighting bones and teeth. The resulting binary slices are then compared to a binary reference mask of a well-aligned lower dental arch (Fig 2a) using the OpenCV correlation coefficient to measure similarity. This coefficient measures how similar two images are, with higher values indicating greater similarity. The highest scoring slice (Figs 2b and S1 for more details) is then morphologically processed. Firstly, the image is *opened*, which reduces noise by removing small artifacts and smoothing out minor imperfections along the edges (Fig 2c). The image is then *closed*, whereupon gaps or inconsistencies in the outline are filled to improve the overall shape of the jaw (Fig 2d). Following this, the image is transformed using the skeletonization algorithm from the Python scikit-image library, resulting in a line along the center of the jaw (Fig 2e). The line’s imperfections are then removed through graph-based optimization, which identifies the longest continuous path (Fig 2f). The center line of the lower jaw is represented by 12 evenly spaced points along its length to capture the overall shape. This number was chosen to capture the overall shape of the jaw accurately, providing enough points to represent the curvature without over- or underfitting. The last two points on each side are used to interpolate an additional point that maintains the same spacing. This provides a more accurate representation of the full length of the jaw (Fig 2g, blue points). The coordinates are shifted 2 cm in the cranial direction along the z-axis, with the intention of moving the lower dental arch away from the center of the image and aligning it more closely with the typical position seen on a PR, where the lower dental arch is not centered (S2a Fig for an example). This position of the coordinates and the resulting PR-like image are referred to as standard in this study.

#### Rotation adjustment.

**Fig 3:**
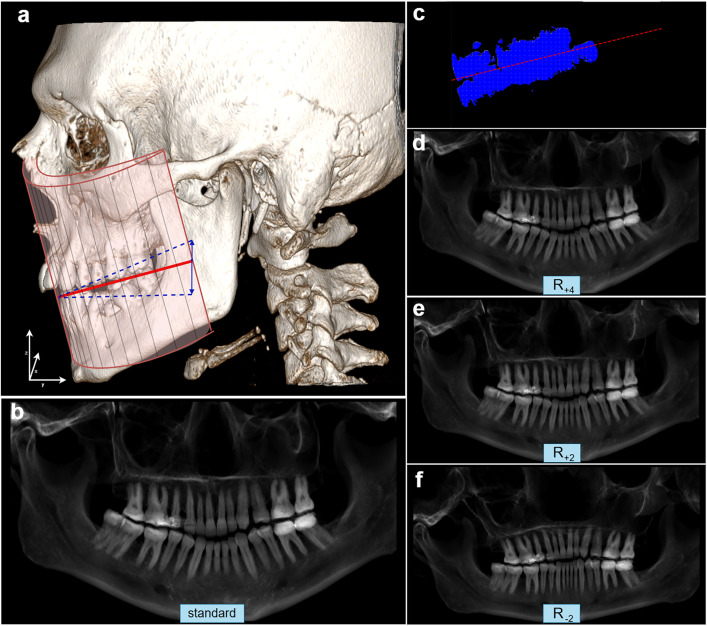
Representation of reconstruction variants of PR-like images. a) Schematic of the 3D volume used for curved MPR, with the resampling volume shown as the pink area outlined in red. The grey lines indicate the spacing of the volume, and therefore the spacing along the vertical axis of the final PR-like image. The volume is tilted according to the red line, showing the base of the rotated reconstruction. Blue arrows indicate changes for the different rotations that were tested. b) Standard reconstruction generated from the initial coordinates (compare with section “Tooth coordinate localization”), before adjustment to the occlusal plane. c) Automated estimation of the occlusal plane in the flattened 3D volume, showing the plane between the upper and lower dental arches in side view (compare with red line in a). The occlusal plane is determined by applying a 3000 HU high-pass filter, projecting the data to a side view, and fitting a regression line to the teeth. The slope derived from this line is applied to the 14 coordinates to adjust the height along the z-axis based on the distance along the y-axis, rendering the resulting image (R_base_, not shown in this figure) independent of head orientation in the CT data. d–f) Additional rotations of the PR-like images are then performed, during which further adjustments of the 14 coordinates are made to fine-tune the height along the z-axis based on the y-axis. Further details can be found in the S1–S3 Figs.

Optionally, the height of these curve-characterizing points can be adjusted to rotate the resulting PR-like image. Initially, the occlusal plane is determined from the CT data by applying a strong high-pass filter (3000 HU) to highlight the teeth region. The data is then flattened along the frontal axis to create a side view. It is then converted into a binary image using OpenCV’s threshold function with a threshold of 0.5, such that pixels greater than 0.5 are set to white and pixels less than or equal to 0.5 are set to black. The blue pixels in Fig 3c represent the teeth of the upper and lower dental arches from the side view, which can be compared to Fig 3a, where the skull is also shown in a side view. A line is fitted to the side-view teeth using linear regression (red line in Fig 3c), which estimates the slope of the occlusal plane. This slope is then used to adjust the vertical position (z-axis) of the 14 defined coordinates. While the height of the frontmost points (incisors) remains unchanged to provide a stable reference for the jaw’s orientation, the positions of the other teeth are shifted up or down along the y-axis depending on the slope, to better match the natural alignment of the jaw (Figs 2g and S2b). The position of the coordinates and the resulting PR-like image are referred to as R_base_ in this study, ensuring that the resulting image is independent of the individual’s head orientation in the CT data.

A controlled rotation is introduced into the PR-like image by defining the rotation parameter R. This parameter modifies the vertical position (z-axis) of the curve-defining coordinates that guide the reconstruction. The adjustment is applied progressively along the jaw. The frontmost point (incisor region) remains unchanged, while the posterior points (towards the molars) are increasingly shifted depending on their position along the y-axis (Figs 2g and S2c). A positive R value (e.g., R = 1, 2, 3,...) lowers the posterior part of the curve, resulting in a forward-tilted appearance of the entire PR-like image. Conversely, a negative value (e.g., R = −1, −2, −3,...) raises the back of the curve, creating a backward tilt. Each increment or decrement of R causes a proportional change in the vertical position of the posterior coordinates (Figs 3d–f, S2 and S3).

To convert these adjusted 3D coordinates into a clinically interpretable PR, the following step generates a 2D PR-like reconstruction from the 3D volume.

#### From 3D volume to 2D PR-like image.

In the subsequent step, the open-source software 3D Slicer (version 5.6.1) [12] and its sandbox extension are used to perform the curved MPR, using the coordinates to generate the curve by b-spline interpolation. A sampling volume (highlighted in red in Fig 3a) is defined along the 14 coordinates, with a height of 10 cm (7 cm above and 3 cm below the curve) and a width of 3 cm (1.5 cm in each lateral direction). This volume is constructed within the original CT volume, which consists of stacked CT slices. The area of included pixels is calculated using the spacing between slices and the pixel spacing within the slices. The volume is then sampled by linear interpolation according to its resolution to achieve a uniform resolution of 0.10 mm/pixel in width and height, and 0.35 mm/pixel in depth.

Following the application of a high-pass filter to the 3D volume to reduce soft tissue and noise, the average intensity is computed along the depth axis of the volume to generate the final 2D PR-like image. This process effectively flattens the structure while preserving key anatomical features, such as the teeth. Prior to the finalization of the PR-like image, an additional optimization step is performed for each non-standard rotation. Initially, the orientation of the teeth within the volume is assessed by extracting the brightest 10% of pixels. Linear regression is then used to determine the angle of deviation. Based on this evaluation, a second curved MPR is generated with the teeth aligned horizontally along the flattened axis. This realignment ensures that the molar edges are emphasized, that distortion is minimized, and that all teeth are included in the volume of the curved MPR, thus further refining the PR-like image. Finally, the resolution of the PR-like image is interpolated, and the image is saved as an 8-bit DICOM file.

### Systematic parameter variation

Three parameters were systematically varied in order to assess their impact on the results: (1) the high-pass filter for reducing soft tissue, with values ranging from 0 to 1500 HU in steps of 250 HU, (2) the interpolated final resolution, which was tested at 0.090, 0.095, 0.100, 0.105, 0.110, 0.115, 0.120, 0.130, and 0.140 mm/pixel, and (3) the rotation parameter R, which was tested with values of +7, + 6,..., base,..., −3, alongside the standard coordinate alignment. In total, 37,800 comparisons (7 x 9 x 12 × 50) were made between the PR-like image and the PR of the same identity across 50 individuals. The objective was to maximize the number of matching points, which are automatically extracted CV features that appear comparable and are matched with one another in both the PR-like image and the reference PR (see next section for more details). An increased number of matching points increases the likelihood of correct identification. The number of matching points was also used to select optimal parameter settings, establishing fixed settings that are suitable for a larger number of cases.

### Identification

Based on the systematic parameter variation, five PR-like images were selected for each of the 50 CT examinations of 50 individuals for comparison with a PR database consisting of 82,036 PRs from 43,379 individuals, using the existing CV-based personal identification method and the settings described by Heinrich [5]. The five PR-like images were generated as follows:

1. Standard coordinate alignment (without additional rotation);
2. R_+4_ (rotation step +4, see section Rotation adjustment);
3. R_+2_ (rotation step +2);
4. R_-2_ (rotation step −2, indicating curvature in the opposite direction, Fig 3);
5. R_best_ (individually optimized combination of parameters yielding the highest number of matching points).

For points 1–4, uniform parameters were applied: a high-pass filter of 500 HU and interpolation with a resolution of 0.115 mm/pixel, both of which were derived from the outcomes of the systematic parameter variation. The rotation steps R_+4_, R_+2_, and R_-2_, along with the standard image, were determined as the best covering set by testing all possible combinations of four rotations and selecting the best-performing image of each individual, thereby ensuring a broad range of potential rotations. This wide range increased the likelihood that one of the rotations would closely match the individual’s actual PR, resulting in a higher number of matching points for accurate personal identification. For point 5, for each individual, the optimal combination of parameters (rotation R, high-pass filter, and interpolation resolution) was selected to achieve the highest possible number of matching points, yielding the PR-like image referred to as R_best_.

The CV-based personal identification method has been comprehensively described in prior publications [4–8]. The following provides a concise summary of the aspects most relevant to the current study.

Prior to CV feature extraction, both the PR-like images and the PRs were preprocessed, including color depth normalization to 8-bit and cropping to a final image size of 180 mm × 100 mm. Edge enhancement was performed using eight Sobel filter masks at orientations from 0° to 315° in 45° steps, with an edge strength modulation parameter of 1.8. The maxima across these gradient images were combined to emphasize edges in all directions, followed by smoothing to reduce image noise. This preprocessing facilitated robust feature extraction using the CV algorithm AKAZE [13,14]. It automatically detects and describes CV features, which identifies keypoints across the entire image, thereby capturing a wide range of anatomical details that extend beyond merely teeth and dental restorations. The algorithm undertakes analysis of image details at multiple scales to detect distinctive points, such as edges and corners. Each CV feature consists of a keypoint along with a descriptor that captures the local image structure. The descriptor acts as a distinctive fingerprint and is robust to transformations such as rotation and scaling. This descriptor serves as a unique representation of the surrounding area around the keypoint, allowing it to be reliably matched across different images, even if the images are rotated, resized, or otherwise transformed. The resulting CV features from PRs were stored in the PR database (S4 Fig), and those from PR-like images were used as queries for comparison.

The identification process involves the comparison of the CV feature set (query set) extracted from a PR-like image with the CV feature sets stored in the PR database, where each PR in the database corresponds to a unique feature set. Matching points are automatically identified when the CV feature descriptors of the compared sets exhibit a high degree of similarity using the squared Euclidean distance. The greater the number of matching points, the higher the probability of correct identification. For further details on the CV algorithm and the matching process, please refer to the references [6,7]. A similarity score ranging from 0% (no match) to 100% (identical images) was calculated by normalizing the number of matching points against the total number of CV features in the PR-like image:


$$score=\frac{{\text{matching}\_\text{points}}_{AB}\;+\;{\text{matching}\_\text{points}}_{BA}}{\text{2}\;\cdot {\;CVfeatures}_{A}}\cdot \text{1}\text{0}\text{0}\;\%$$ (1)

The matching process was performed twice: first by comparing the query set (A) with a database entry (B) and then in reverse (B to A). The final score was calculated as the average number of matching points from both directions, ensuring a more robust comparison by accounting for both orientations.

The identification rate evaluates the accuracy of recognizing individuals. Rank 1 indicates that the score for images of the same identity is the highest among all database entries. In rank 10 and rank 100, the score for the correct individual is among the top 10 or top 100 highest scores, respectively. To further enhance identification performance, a combined result was calculated by averaging the two highest scores from the four PR-like images (standard, R_+4_, R_+2_, and R_-2_) for each identity. Statistical significance (α = 0.05) was assessed using the Mann-Whitney U test to compare score distributions between groups (same identity vs. different identities), implemented using Python’s SciPy library.

## Results

### Parameter evaluation

**Fig 4:**
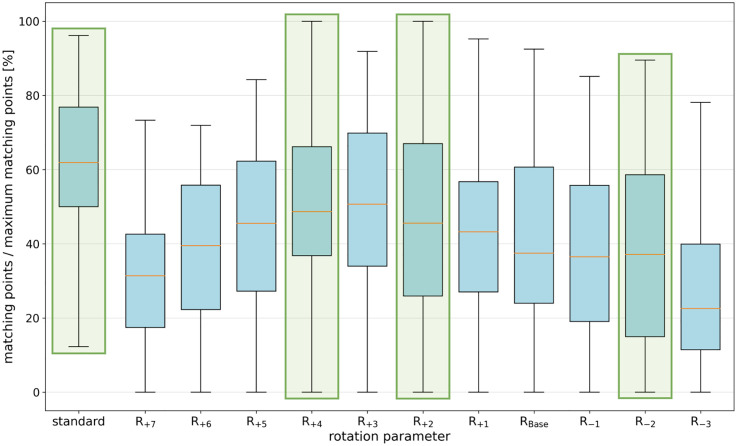
Performance of variations of the rotation parameter. For CV-based comparisons of PR-like images with the PRs of the same identity, the number of matching points for different reconstructions R (with a high-pass filter of 500 HU and interpolated resolution of 0.115 mm/pixel) is shown and normalized based on the maximum number of matching points resulting from the optimal parameter configuration R_best_. The chosen parameters used in the combined method are highlighted in green.

As illustrated in Table 1, the results of the systematic variation of high-pass filtering and resolution are shown, with the average number of matching points across all 12 rotations of the PR-like images for each of the 50 identities. It was determined that a combination of 500 HU for the high-pass filter and 0.115 mm/pixel for the resolution yielded the maximum number of matching points. Furthermore, Fig 4 shows the results for a fixed high-pass filter (500 HU) and resolution (0.115 mm/pixel) across different rotations in the curved MPR. The standard reconstruction and three additional reconstructions (R_+4_, R_+2_, and R_-2_) were selected for subsequent matching with a large PR database. The rationale behind the selection of these specific rotation adjustments was to encompass a wide spectrum of curvatures, thereby enhancing the likelihood of alignment with the PR images of the individual to be identified in the database, and thereby increasing the robustness of the identification process.

**Table 1:** Average matching points depending on interpolated resolution and high-pass filter.

high-pass filter [HU]	interpolated resolution [mm/pixel]								
	0.090	0.095	0.100	0.105	0.110	**0.115**	0.120	0.130	0.140
0	5.84	9.44	9.92	10.63	10.88	10.66	9.89	7.80	6.18
250	6.00	9.85	10.59	11.43	11.66	11.59	10.86	8.82	7.08
**500**	5.99	9.90	10.50	11.17	11.70	11.70	11.12	9.12	7.29
750	5.72	9.24	9.75	10.47	10.88	11.10	10.61	8.99	7.18
1000	5.26	8.50	8.92	9.32	9.74	10.06	9.92	8.34	6.61
1250	4.63	7.32	7.68	8.08	8.56	8.80	8.79	7.60	5.98
1500	3.82	6.05	6.32	6.66	7.06	7.19	7.37	6.55	5.16

### Outcomes of identification

**Fig 5:**
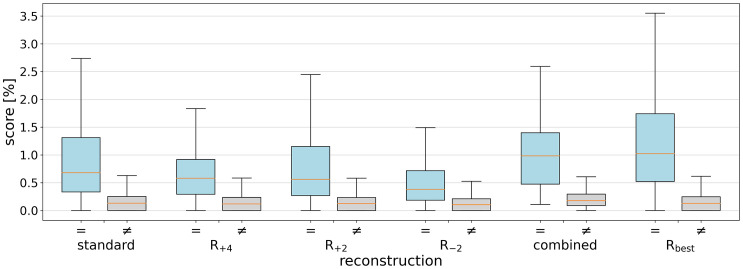
Comparison of matching scores across reconstructions for same versus different individuals. The boxplots show the scores (number of matching points divided by the total number of CV features in the query image) for the different reconstructions R against the PR database. The blue bars represent the scores for comparisons within the same individuals (=), while the grey bars represent comparisons between different individuals (≠).

The standard PR-like image achieved an identification rate of 19/50 (38%) at rank 1 (where the sought person has the most matching points), 31/50 (62%) at rank 10, and 33/50 (66%) at rank 100, with a potential of 43,379 individuals (Table 2). The PR-like images generated with different rotations based on the occlusal plane generally have lower identification rates, but they could achieve higher scores for specific individuals. This variation was due to the differing representation of tooth edges, which can influence the CV features extracted from the images. By combining four different PR-like images from the same CT data (using the mean of the highest two scores for each identity), the identification rate improved, reaching 25/50 (50%) at rank 1, 32/50 (64%) at rank 10, and 39/50 (78%) at rank 100. When the optimal parameter setting (R_best_), determined from the systematic variation, was applied to each identification case, an identification rate of 36/50 (72%) at rank 1, 41/50 (82%) at rank 10, and 48/50 (96%) at rank 100 is achieved. A comparison of PR-like images with PRs from the same individual resulted in a higher number of matching points, averaging 0.91 ± 0.76% (with 100% representing the maximum possible matching points), while images from different individuals averaged 0.17 ± 0.20% for the standard PR-like image (Fig 5). A further comparison of the scores between same identity and different identities resulted in a p-value of < 0.001, indicating a highly significant difference between these groups.

**Table 2:** Identification rates and matching scores for different PR-like images.

PR-like image	score [%]		identification rate [%]			
	MW (=)	MW (≠)	rank 1	rank 10	rank 100	rank 1000
standard	0.91 ± 0.76	0.17 ± 0.20	38	62	66	88
R_ + 4_	0.70 ± 0.60	0.15 ± 0.17	22	34	54	68
R_ + 2_	0.79 ± 0.70	0.16 ± 0.17	28	38	60	76
R_-2_	0.66 ± 1.12	0.14 ± 0.16	22	30	46	60
combined	1.06 ± 0.88	0.21 ± 0.19	50	64	78	92
R_best_	1.30 ± 1.25	0.17 ± 0.18	72	82	96	98

The table shows the identification rates for 50 cases using various reconstructions of PR-like images, matched against a PR database containing 82,036 PRs from 43,379 individuals. Additionally, results for the combined approach (standard, R_+4_, R_+2_, and R_-2_) and the best individual parameter setting (R_best_) are shown. Furthermore, the scores (number of matching points divided by the total number of CV features in the query image) for comparisons within the same individuals (=) and between different individuals (≠) are presented, highlighting that the same identity shows more matching points.

**Fig 6:**
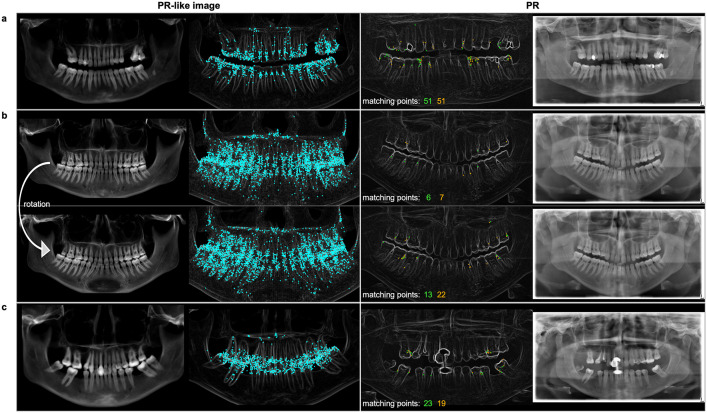
Overview of CV feature matching between PR-like image and PR of the same identity. Blue points show CV features found in PR-like image, green points indicate matches from the PR to the PR-like image (BA), while orange points represent matches from the PR-like image to the PR (AB). Tooth edges provide a multitude of distinct matching points, a) especially when the mouth is slightly open or b) when the PR-like image is well-aligned with the PR in terms of rotation. Furthermore, c) dental fillings can also be detected. The enhanced visibility of contours in columns 2 and 3 results from the edge-enhancing preprocessing applied to the PR-like image and the reference PR.

Fig 6 shows a comparison between the PR-like image and the PR image, including the CV features from the PR-like image (represented as blue points) and the matching points found in the PR of the same identity (represented as orange points from PR-like image to PR, and green points from PR to PR-like image). Specifically, the edges of the teeth contain unique CV features (a), which facilitate precise identification but can be concealed by tooth overlap (b, c). The degree of tooth overlap affected the identification rate. Among the 50 cases examined, 13 exhibited substantial tooth overlap, resulting in a 23% identification rate at rank 1. In 17 cases with partial overlap, the identification rate increased to 47%, while the highest identification rate of 70% was observed in 20 cases with clear separating edges between all teeth. Furthermore, it was observed that adjusting the rotation of the PR-like image reconstruction can lead to a substantial increase in the number of matching points (b). Moreover, the identification rate based on the spacing between slices in the CT data was evaluated. At rank 1, the identification rate was 54% (15/28) for the 0.3125 mm spacing and 45% (10/22) for the 0.625 mm spacing. See S1 Table for further details.

The average runtime per PR-like image for a complete PR database comparison was 33.89 ± 8.91 min (median 32.79 min) on a standard desktop computer (Intel i5-8500 CPU @ 3.00 GHz, 16 GB RAM, no hardware acceleration).

## Discussion

In this retrospective study, the feasibility of CV-based personal identification was tested by matching PR-like multiplanar reconstructions from multidetector CT with PRs. PR-like images from 50 randomly selected CT scans were compared with a large clinical PR database containing 82,036 PRs from 43,379 individuals. Rank 1 identification was achieved in 38% of cases using a standard reconstruction, 50% when combining four fixed reconstructions, and 72% with individually optimized parameters (R_best_). These results highlight the critical impact of reconstruction settings on cross-modality identification performance. In principle, one PR-like image and a corresponding reference PR can enable successful identification, even in a large-scale dataset, if their image representations are sufficiently comparable (S1 Table).

Few previous studies [9–11] have examined the comparability of PR-like images generated from multidetector CT with PRs, and none have implemented a fully automated workflow with large-scale database comparisons as presented in this study. Fujimoto et al. [10] compared PR-like images of two living individuals with 100 reference PRs using Procrustes analysis and correlation coefficients, confirming identity through a six-segment method. Although this study demonstrated the feasibility of PR-like image comparison, it was limited to very small datasets and manual verification. Tohnak [9] and Vincent et al. [11] also demonstrated that CT data can be used to generate curved MPRs suitable for forensic comparison by experts. While these studies confirmed that PR-like images can resemble PRs, they did not explore automated identification or systematically evaluate factors such as dental overlap or image resolution, as addressed in the present study. Additionally, other imaging modalities have been explored for PR-like image generation. Eliasova et al. [15] compared cone beam PR-like images with PRs and found both modalities complementary for forensic purposes, showing high correlation and a mean image coincidence of 6.1%. However, these comparisons were performed manually by two observers with visual inspection and point-based image registration, rather than via an automated process. Similarly, PR-like images derived from magnetic resonance imaging (MRI) [16,17] demonstrated no significant quantitative differences compared with PRs. However, these comparisons were based on manually reconstructed PR-like images from various MRI sequences and evaluated by human readers, without any automated processing. Furthermore, metallic materials such as fillings or implants could cause artifacts affecting image quality [18,19]. In contrast to previous approaches limited to manual verification or small datasets, the present study demonstrates fully automated generation of PR-like images from multidetector CT and their use for automated identification of individuals through CV-based comparison with clinical PR databases.

Automatic PR-like image generation from multidetector CT data is rarely addressed in the literature. A recent review [20] identified only a few contributions, among which Sa-ing et al. [21] proposed a semi-automatic method requiring manual selection of a representative CT slice, while Han et al. [22] developed a fully automatic approach based on maximum intensity projection (MIP) and medial axis fitting. These methods typically involved arch detection, curve fitting along a reference, and projection to synthesize PR-like images. The present study shares the core steps of arch detection, curve fitting, and projection. It differs in several key aspects. It performs fully automatic curved MPR from the 3D CT volume, allows flexible rotation adjustments, and optionally optimizes teeth alignment. Fully automated methods are more common for cone beam CT [23–26], but their adaptation to multidetector CT is limited due to differences in acquisition geometry and image properties. Moreover, previous studies typically generated only a single PR-like image per dataset, or at most minor offsets along the detected dental arch [21,23]. In contrast, the present study systematically generates multiple PR-like variants from the same CT dataset to improve the matching with unknown PRs in cross-modality CV-based personal identification.

The reconstruction geometry, particularly the virtual rotation during curved MPR, strongly influenced identification accuracy. Because the exact orientation of the dental arch in the reference PR is unknown and varies between individuals, multiple reconstruction variants increase the likelihood of achieving good alignment. In this study, combining four fixed variants significantly improved rank 1 rates. In 40 of 50 cases, the empirically optimal reconstruction (R_best_) used a rotation different from the standard, confirming the sensitivity of the method to reconstruction geometry. Adjusting rotation could substantially increase the number of matching CV features and thus improve identification reliability (Fig 6b). High-pass filtering also influenced performance. It enhanced the contrast of dense structures such as teeth and implants by suppressing lower-density regions. Moderate thresholds (around 500 HU) offered an optimal trade-off between contrast enhancement and information preservation. Excessive filtering, by contrast, removed useful details and reduced the number of matching features. Similarly, when the interpolated resolution of the PR-like image approximated that of the clinical PR, the number of matching points increased. The results with R_best_ illustrate the achievable upper limit of the current method, showing that individualized optimization of reconstruction parameters can markedly improve performance beyond fixed settings. The lower identification rates observed with fixed parameter settings (e.g., 50% at rank 1 with the combined approach) highlight the limitations of a one-size-fits-all reconstruction strategy. While fixed parameters enable a fully automated and computationally efficient workflow, they cannot account for every anatomical variation, limiting rank 1 identification success. Individually optimized reconstructions (R_best_) require prior knowledge or extensive computational resources to test multiple variants, making them impractical for large-scale automated applications. Future work could explore adaptive reconstruction strategies or automated parameter selection based on preliminary image analysis to mitigate these limitations.

**Fig 7:**
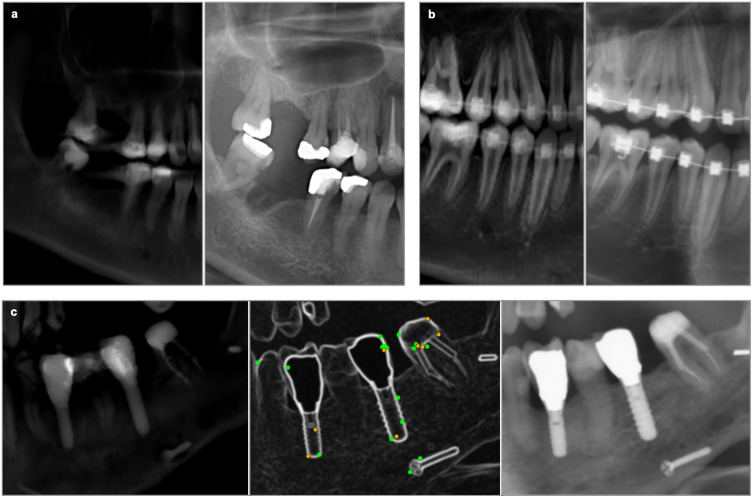
Examples of cropped sections from PR-like images (left) and PRs (right). Showing a) dental fillings, b) braces, and c) implants, illustrating the contrast differences between the modalities. In c), CV matching points (green and orange) demonstrate that, despite the lower contrast in the PR-like image, matching points were still identified along the edges of the dental implants.

Anatomical and acquisition-related factors contributed to variability in matching success. Dental restorations appeared with lower contrast in PR-like images compared to PRs (Fig 7). While restorations can provide distinct CV features in PRs [4], they were less consistently detected in PR-like images, although implant edges sometimes yielded usable matches (Fig 7c). Tooth overlap during CT acquisition also had a marked impact. Because patients typically undergo CT scanning with closed jaws and without a bite block, occlusal edges are often obscured. The degree of tooth separation correlated with identification rates: 23% for strong overlap (13 cases), 47% for partial overlap (17 cases), and 70% for clear separation (20 cases). Slice spacing also affected results, with higher resolution (0.3125 mm) yielding better performance (54% vs. 45%). These findings underscore the importance of acquisition geometry and image quality for cross-modality matching.

Metal artifacts represent a major limitation [9]. They obscure dental contours and restorations, which are critical for CV-based matching. Consequently, CT datasets with pronounced artifacts were excluded from this study. Both PR-like reconstruction and CV-based matching are fully automated and deterministic. Repeated analyses with identical inputs and parameters always yield identical PR-like images and matching scores, ensuring full reproducibility. Classical reproducibility metrics such as Cohen’s kappa are therefore not applicable, as no variability is introduced by human observers or stochastic processes. Nonetheless, the reliability of the approach depends on reconstruction quality and anatomical comparability, which may change over time due to growth, dental treatment, or trauma.

The use of AKAZE, a classical CV algorithm, enables the extraction of multiscale, rotation- and scale-invariant features across the entire image. These features differ from traditional forensic odontological characteristics used in expert-based identification [27,28], such as tooth count, restoration patterns, and implant configurations. Consequently, CV-based results are not legally sufficient for identification but can serve as a preselection tool to narrow down candidate sets and support expert-based verification. Automated large-scale matching could therefore accelerate the identification process in forensic or disaster victim identification contexts, where time and data volume are critical.

This study demonstrates a proof of concept that CV-based personal identification across modalities, specifically between PR-like images and PRs, is feasible. However, several limitations should be acknowledged. Although an automatic algorithm for generating PR-like images was developed and systematically evaluated, further refinements, such as enhancing contrast for dental restorations or optimizing CV method parameters, remain areas for future research. Additionally, incorporating demographic factors like gender and age [5] could further improve accuracy. Moreover, a systematic assessment of the effect of metal artifacts on identification and developing strategies to reduce their influence represents an important direction for future research.

## Conclusions

Automated generation of PR-like images from multidetector CT, combined with CV-based personal identification using a large clinical PR database, demonstrates the technical feasibility of cross-modality identification. Using multiple PR-like variants per individual and adjusting reconstruction parameters, particularly rotation, significantly improves identification rates: 50% of individuals were correctly identified at rank 1 using a combined approach, increasing to 72% when individually optimized parameters (R_best_) were applied. These results highlight the critical impact of reconstruction settings on identification performance and underscore the potential of this approach as a rapid, scalable tool to support forensic or emergency workflows. Future improvements in imaging resolution and dental structure representation are expected to further enhance reliability and applicability.

## Supporting information

S1 Fig Extended view of CT slice selection used for curve extraction. This figure illustrates the process of extracting 14 coordinates from binarized CT images within the series. a) CT images are annotated with slice indices. b) A correlation coefficient is used to quantify the similarity between the CT images and a lower dental arch mask; slice index 138 has the highest correlation coefficient. c) Slice 138 is further processed using morphological filtering: an open operation removes small-scale noise, followed by a close operation that fills gaps and smooths the jaw contour. The resulting image is then skeletonized to generate a centerline of the lower jaw. Protruding branches are removed by graph-based optimization. Along the optimized curve, 12 evenly spaced coordinates are placed, and an additional coordinate is appended to each end of the curve (shown as blue dots), for a total of 14 coordinates.
(TIF)

S2 Fig Illustration of modifications to the 14 coordinates and the resulting changes in the reconstructed images. Starting with the 14 coordinates derived from the lower dental arch (black points in a), further adjustments are applied. First, the coordinates are shifted 2 cm cranially along the z-axis to resemble the center of the panoramic radiograph and therefore better fit its natural scope (see red points and PR-like image). In b), the occlusal plane is estimated by applying a high-pass filter to the CT data and projecting the result onto a side view. Blue pixels represent the dental arches; a linear regression (red line) is fitted to extract the slope. This slope is used to vertically adjust the 14 coordinates, defining the base configuration R_base_ (see green points and the PR-like image in b). In c), further z-axis modifications are applied to simulate different rotations. Green points indicate R_base_, yellow points show examples of rotated configurations (compare with PR-like images).
(TIF)

S3 Fig Examples of the systematic variation of three parameters. Variation of the rotation parameter (R), interpolated resolution, and high-pass filter strength in the PR-like image generation is shown. In addition, the PR-like image generated with the optimal parameter set R_best_, representing the combination of parameters that yielded the highest number of matching points with the reference PR image, is presented.
(TIF)

S4 Fig Distribution of the PR database. The table shows the distribution of 82,036 PRs in the database by age and gender, based on acquisition dates between 2002 and 2023.
(TIF)

S1 Table Overview of the 50 test cases used in the study. This table summarizes demographic and imaging information for the 50 study cases, including anonymized case number, gender, age group, CT slice spacing, tooth overlap in CT imaging, grouped numbers of teeth, small fillings, crowns/large fillings, dental implants/orthodontic appliances, identification results (comb. and R_best_), and number of reference PRs in the database. Tooth overlap refers exclusively to overlapping dental structures observed in the CT reconstructions and is a technical artifact, not a clinical or anatomical feature.
(PDF)
