# Supplementary figures and images for: Personal identification via matching of curved multiplanar computed tomography reconstructions and panoramic radiographs
Source: PLoS One. 2025 Dec 4;20(12):e0337989. doi: 10.1371/journal.pone.0337989 (PMC12677444; doi:10.1371/journal.pone.0337989)

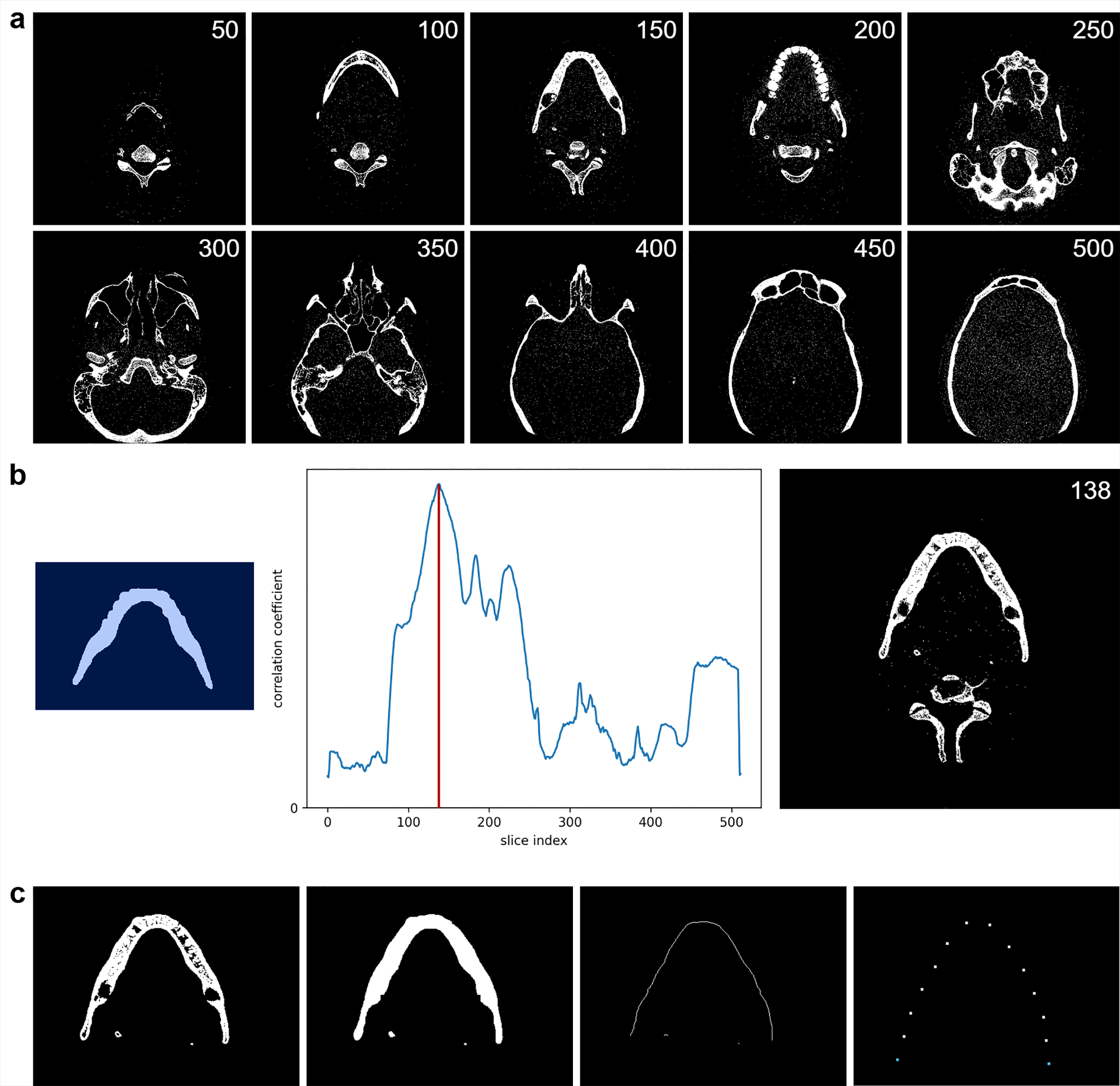

Supplement: S1 Fig — This figure illustrates the process of extracting 14 coordinates from binarized CT images within the series. a) CT images are annotated with slice indices. b) A correlation coefficient is used to quantify the similarity between the CT images and a lower dental arch mask; slice index 138 has the highest correlation coefficient. c) Slice 138 is further processed using morphological filtering: an open operation removes small-scale noise, followed by a close operation that fills gaps and smooths the jaw contour. The resulting image is then skeletonized to generate a centerline of the lower jaw. Protruding branches are removed by graph-based optimization. Along the optimized curve, 12 evenly spaced coordinates are placed, and an additional coordinate is appended to each end of the curve (shown as blue dots), for a total of 14 coordinates. (TIF) [file pone.0337989.s001.tif]

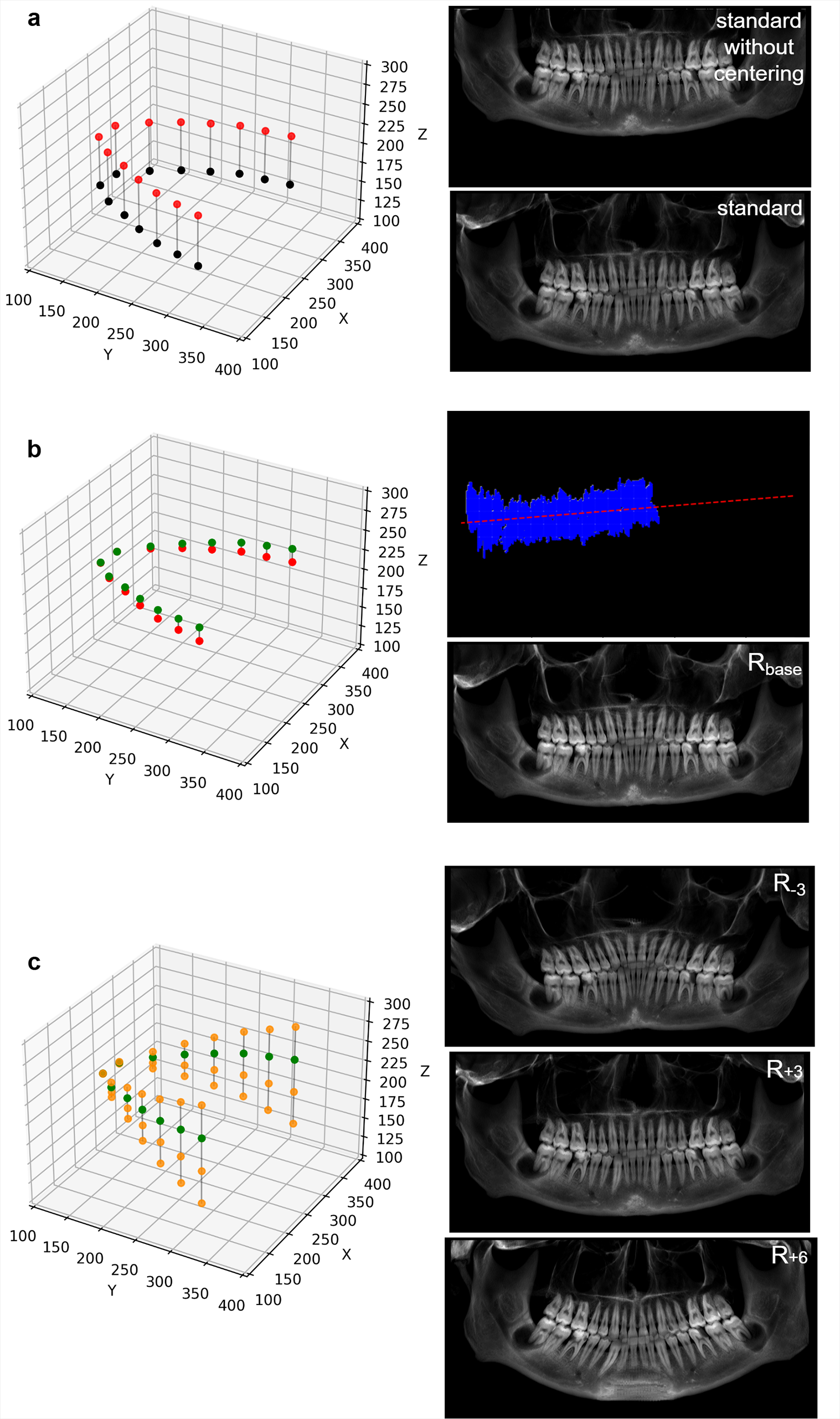

Supplement: S2 Fig — Starting with the 14 coordinates derived from the lower dental arch (black points in a), further adjustments are applied. First, the coordinates are shifted 2 cm cranially along the z-axis to resemble the center of the panoramic radiograph and therefore better fit its natural scope (see red points and PR-like image). In b), the occlusal plane is estimated by applying a high-pass filter to the CT data and projecting the result onto a side view. Blue pixels represent the dental arches; a linear regression (red line) is fitted to extract the slope. This slope is used to vertically adjust the 14 coordinates, defining the base configuration Rbase (see green points and the PR-like image in b). In c), further z-axis modifications are applied to simulate different rotations. Green points indicate Rbase, yellow points show examples of rotated configurations (compare with PR-like images). (TIF) [file pone.0337989.s002.tif]

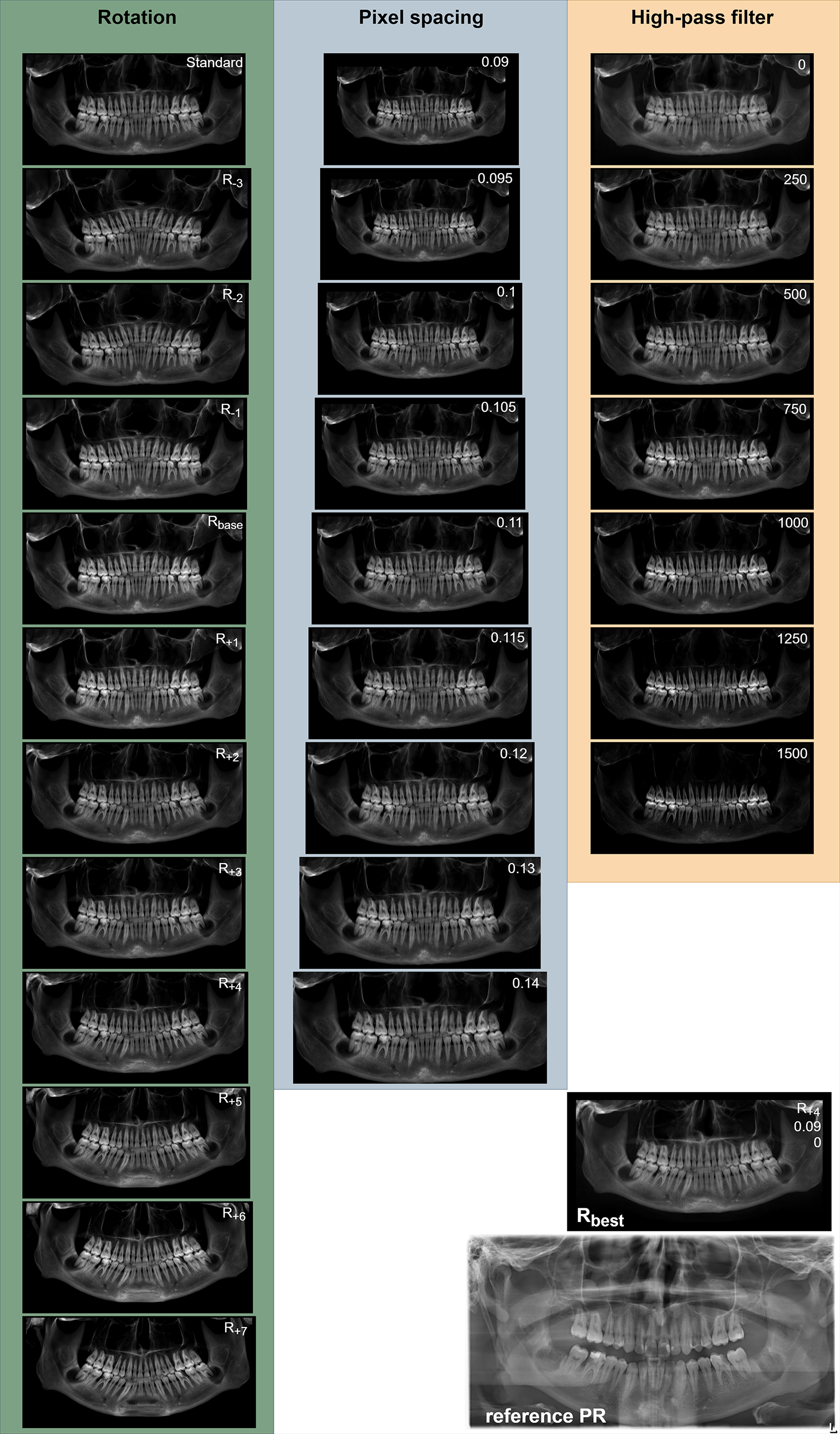

Supplement: S3 Fig — Variation of the rotation parameter (R), interpolated resolution, and high-pass filter strength in the PR-like image generation is shown. In addition, the PR-like image generated with the optimal parameter set Rbest, representing the combination of parameters that yielded the highest number of matching points with the reference PR image, is presented. (TIF) [file pone.0337989.s003.tif]

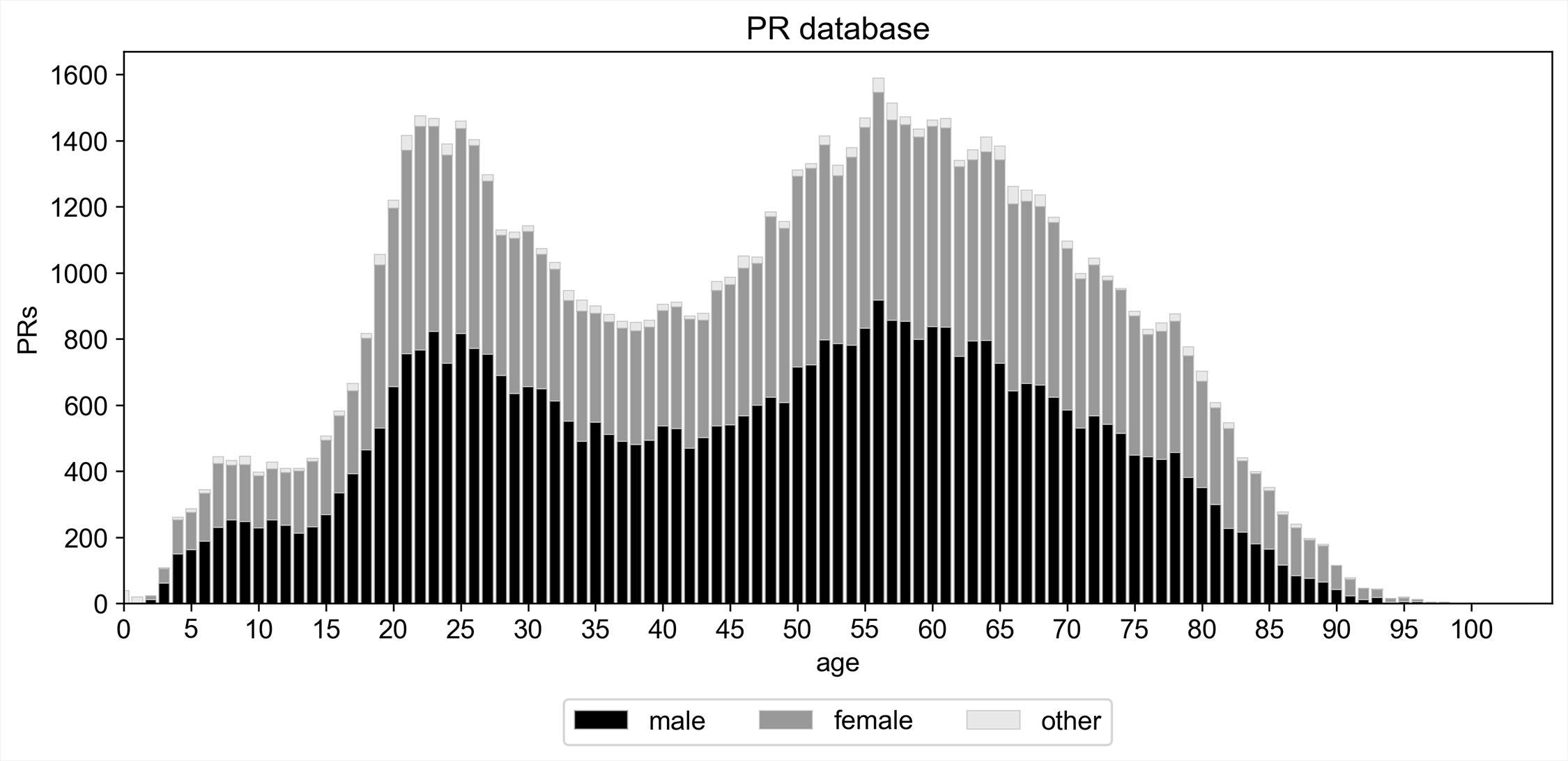

Supplement: S4 Fig — The table shows the distribution of 82,036 PRs in the database by age and gender, based on acquisition dates between 2002 and 2023. (TIF) [file pone.0337989.s004.tif]
