# Supplementary material for: Personal identification via matching of curved multiplanar computed tomography reconstructions and panoramic radiographs
Source: PLoS One. 2025 Dec 4;20(12):e0337989. doi: 10.1371/journal.pone.0337989 (PMC12677444; doi:10.1371/journal.pone.0337989)
Supplement: S1 Table — This table summarizes demographic and imaging information for the 50 study cases, including anonymized case number, gender, age group, CT slice spacing, tooth overlap in CT imaging, grouped numbers of teeth, small fillings, crowns/large fillings, dental implants/orthodontic appliances, identification results (comb. and Rbest), and number of reference PRs in the database. Tooth overlap refers exclusively to overlapping dental structures observed in the CT reconstructions and is a technical artifact, not a clinical or anatomical feature. (PDF) [file pone.0337989.s005.pdf]

S1 Table. Overview of the 50 test cases used in the study.

Case No.	CT examination								results rank		ref. PRs
	gen der	age group [years]	slice spacing [mm]	tooth overlap (imaging)	teeth group	small filling group	crown/ large filling	dental implant/ braces	comb.	R_{best}	
1	F	13-24	0.3125		29-32				1	1	>5
2	M	13-24	0.3125	partial	29-32			yes	335	30	1
3	M	13-24	0.3125	yes	29-32	1-3			9	3	>5
4	M	13-24	0.3125	yes	29-32	1-3			294	2	1
5	F	13-24	0.3125		25-28			yes	435	14	2-5
6	M	13-24	0.3125	partial	29-32	1-3			1	1	2-5
7	M	13-24	0.625		25-28	1-3			300	18	2-5
8	M	13-24	0.625	partial	25-28	1-3			1	1	1
9	M	13-24	0.625	partial	25-28				5	34	2-5
10	M	25-34	0.3125	yes	29-32	1-3			1	1	2-5
11	F	25-34	0.625	partial	25-28	4-6	yes		1	1	1
12	M	25-34	0.625	yes	25-28	1-3			93	1	1
13	M	25-34	0.3125		25-28	1-3			1511	282	1
14	F	25-34	0.3125	partial	25-28	1-3		yes	1	1	2-5
15	M	25-34	0.625		29-32	1-3			1	1	2-5
16	M	25-34	0.625		29-32	4-6	yes		1	1	2-5
17	M	25-34	0.625	yes	25-28	1-3			3	1	1
18	F	25-34	0.3125	partial	25-28	1-3	yes		1	1	2-5
19	M	25-34	0.625		29-32	10-12			51	1	2-5
20	M	25-34	0.3125	yes	25-28	1-3			51	1	2-5
21	M	25-34	0.625		25-28				9101	31	1
22	F	25-34	0.625	partial	25-28	1-3			83	4	1
23	M	25-34	0.3125	yes	25-28	1-3			20	6	1
24	F	25-34	0.3125	yes	25-28	1-3			7	1	1
25	F	25-34	0.625	partial	25-28	4-6	yes	yes	1	1	2-5
26	M	35-54	0.3125	yes	29-32	1-3			2212	23	1
27	M	35-54	0.625	yes	25-28				968	12	1
28	M	35-54	0.3125	partial	21-24				4767	4008	1
29	M	35-54	0.625		12-20	1-3			1	1	1
30	M	35-54	0.625		12-20	4-6			1	1	>5
31	F	35-54	0.3125	yes	25-28	7-9	yes		1	1	2-5
32	M	35-54	0.3125	partial	21-24	1-3	yes		1	1	2-5
33	M	35-54	0.625	partial	29-32	4-6			27	1	2-5
34	M	35-54	0.3125		29-32	7-9	yes		2	1	2-5
35	M	35-54	0.625	partial	25-28	4-6			19	5	1
36	M	35-54	0.3125		25-28	4-6			1	1	1
37	F	35-54	0.625		21-24	7-9	yes		1	1	2-5
38	F	35-54	0.3125		12-20	1-3	yes		1	1	>5
39	M	35-54	0.3125		29-32	1-3	yes		1	1	>5
40	M	35-54	0.3125		25-28	1-3	yes	yes	1	1	2-5
41	F	35-54	0.3125		25-28	4-6	yes		1	1	2-5
42	F	55-82	0.3125		25-28	4-6			1	1	2-5
43	M	55-82	0.625	partial	25-28	1-3			274	1	1

44	F	55-82	0.3125		12-20	1-3		yes	1	1	2-5
45	M	55-82	0.3125	partial	25-28	7-9	yes		232	1	>5
46	M	55-82	0.3125	yes	25-28	4-6	yes		1	1	>5
47	M	55-82	0.625	partial	12-20	1-3	yes		1	1	2-5
48	F	55-82	0.3125	yes	29-32	10-12	yes		2	1	1
49	F	55-82	0.625		29-32	1-3			1	1	1
50	F	55-82	0.625	partial	12-20	1-3	yes		9	1	2-5

This table summarizes demographic and imaging information for the 50 study cases, including anonymized case number, gender, age group, CT slice spacing, tooth overlap in CT imaging, grouped numbers of teeth, small fillings, crowns/large fillings, dental implants/orthodontic appliances, identification results (comb. and R_{best}), and number of reference PRs in the database. Tooth overlap refers exclusively to overlapping dental structures observed in the CT reconstructions and is a technical artifact, not a clinical or anatomical feature.
